# Neonatal pyocele originating from a urinary tract infection: a case report

**DOI:** 10.1186/s13256-024-04416-3

**Published:** 2024-02-28

**Authors:** Zahra Jamali, Mohammad Shafie’ei, Najmeh Soltani Nejad

**Affiliations:** 1 Department of Pediatrics, Afzalipour Faculty of Medicine, Kerman University of Medical Sciences, Kerman, Iran; 2 Faculty of Medicine, Kerman University of Medical Sciences, Kerman, Iran; 3 Department of Pediatrics, Kerman University of Medical Sciences, Kerman, Iran

**Keywords:** Scrotum, Testis, Newborn, Infections, Case reports, Urinary tract infections

## Abstract

**Background:**

The origins of pyocele are primarily idiopathic, with reports suggesting the spread from intraperitoneal or hematogenous infection. However, we found no information in the literature regarding the pathogen’s spread from the urinary tract.

**Case presentation:**

We report here a case of a 23-day-old term Iranian boy (Fars ethnicity) with complaints of new-onset fever, irritability, poor feeding, right hemiscrotal erythema, and edema. Moreover, the physical examination revealed an enlarged, erythematous, tender right hemiscrotum and edematous scrotal walls. Along with leukocytosis and elevated C-reactive protein levels, the urine culture indicated an infection with *Escherichia coli*. However, as the color Doppler ultrasonographic examination was inconclusive, he underwent surgical exploration on which a hydrocele sac with reddish cloudy pus fluid was noted, with its culture indicating growth with the same mentioned pathogen. Therefore, an appropriate antibiotic regimen was administered, and the patient was discharged a few days later after achieving full recovery and demonstrating no urinary tract structural abnormalities.

**Conclusion:**

In neonatal pyocele, the spectrum of evaluating the source of the infection should also be extended to the urinary tract. Moreover, administering suitable antibiotics would produce favorable results in cases with no structural abnormalities.

## Background

Pediatric scrotal edema and its subcategory of scrotum-related emergencies (that is, acute scrotum) are a considerable concern in clinical practice, several of which necessitate early diagnosis and intervention [1]. Although other and more critical conditions, including testicular torsion, need to be considered in such presentations, one rare differential diagnosis is the infected hydrocele or pyocele, requiring emergent antibiotic therapy, and in some cases, surgical exploration with drainage or aspiration [2–4]. Though many are idiopathic, reports suggest the spread from an intraperitoneal infection (that is, via a patent process vaginalis) as the most common etiology [5–8]. Moreover, the pathogens in most reported cases were found to be bacterial, primarily *Escherichia coli* (*E. coli*) and, in some instances, *Staphylococcus lugdunensis*, *Klebsiella pneumonia*, β-hemolytic streptococci, *Salmonella* spp., *Bacteroides fragilis*, and *Proteus mirabilis* [2–6, 9]. However, we found no reports in the literature of the spread of the infection from the urinary tract [3, 9, 10]. Therefore, we report a case of a 23-day-old boy with pyocele and a confirmed urinary tract infection.

## Case presentation

A 23-day-old term Iranian boy (Fars ethnicity) delivered via Cesarean section with an optimal Apgar score and weight of 2800 g (current weight 3900 g) from a 22-year-old mother with a normal pregnancy course was referred to our care center with complaints of new-onset fever, irritability, poor feeding, right hemiscrotal erythema and edema, and bilateral erythematous inguinal lumps starting 24 h before admission. He had a history of uninvestigated neonatal jaundice on the third day after birth, albeit with desirable weight gain and no complaints of poor feeding.

**Fig. 1:**
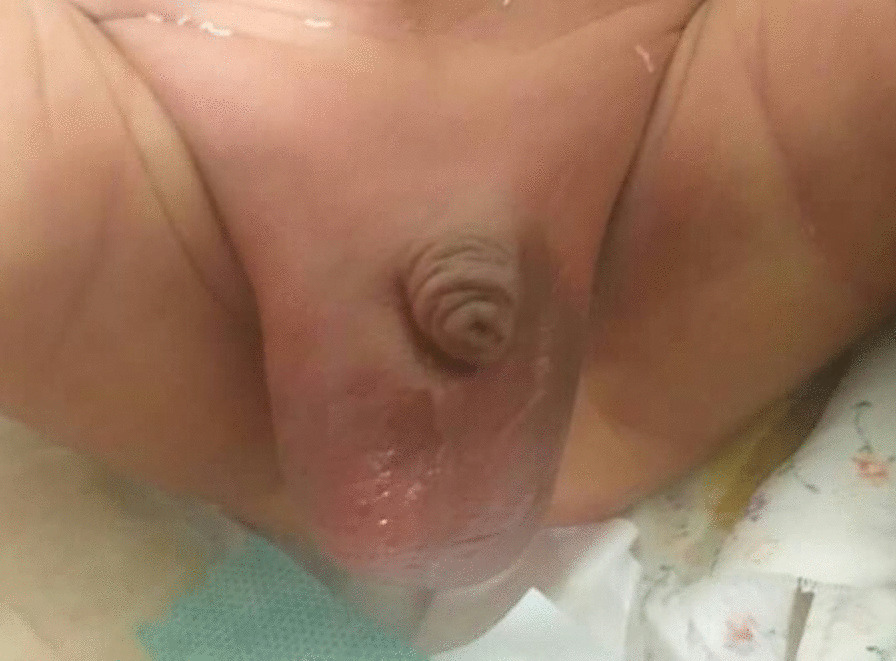
Scrotum appearance on admission

The initial physical examination revealed a well-nourished, alert, generally icteric infant with vital signs in normal ranges except the core temperature (higher than the normal range) (Table 1). Neonatal reflexes were present on evaluation, while no abnormal findings were found on the examination of the head, neck, chest (including heart and lungs), and abdomen (soft and nontender). However, an enlarged, erythematous, tender right hemiscrotum and edematous scrotal walls and bilaterally erythematous and bulged inguinal area (more pronounced on the right side) were notable (Fig. 1). Therefore, the patient was admitted with empirical intravenous ampicillin (50 mg/kg/dose three times daily) and amikacin (15 mg/kg once daily) due to suspicions of ongoing sepsis.

**Table 1:** Data obtained from the patient’s physical (that is, vital signs) and laboratory examinations

Index	Value		
*Vital signs*			
Heart rate	178 beats per minute		
Respiratory rate	44 breaths per minute		
Core temperature	38.8 °C		
Blood oxygen saturation	96%		
*Laboratory data*			
White blood cell count and their differentiation	15,700	Neut%	55%
		Lymph%	34%
Hemoglobin	13.5 mg/dL		
Platelet count	444,000		
Total bilirubin	14 mg/dL		
C-reactive protein	18 mg/dL		
Cultures			
Cerebrospinal fluid	No growth		
Urine	*Escherichia coli*		
Blood	No growth		
Hydrocele fluid	*Escherichia coli*		

*Neut* neutrophils, *Lymph* lymphocytes

The laboratory investigations revealed leukocytosis and elevated C-reactive protein (CRP) levels, meaning inflammation was present. Blood, urine, and cerebrospinal fluid (CSF) cultures were also obtained, with only that of the urine indicating *E. coli* growth (antibiogram revealing susceptibility to ampicillin and amikacin) and others returning negative for any pathogenic organism growth (Table 1).

**Fig. 2:**
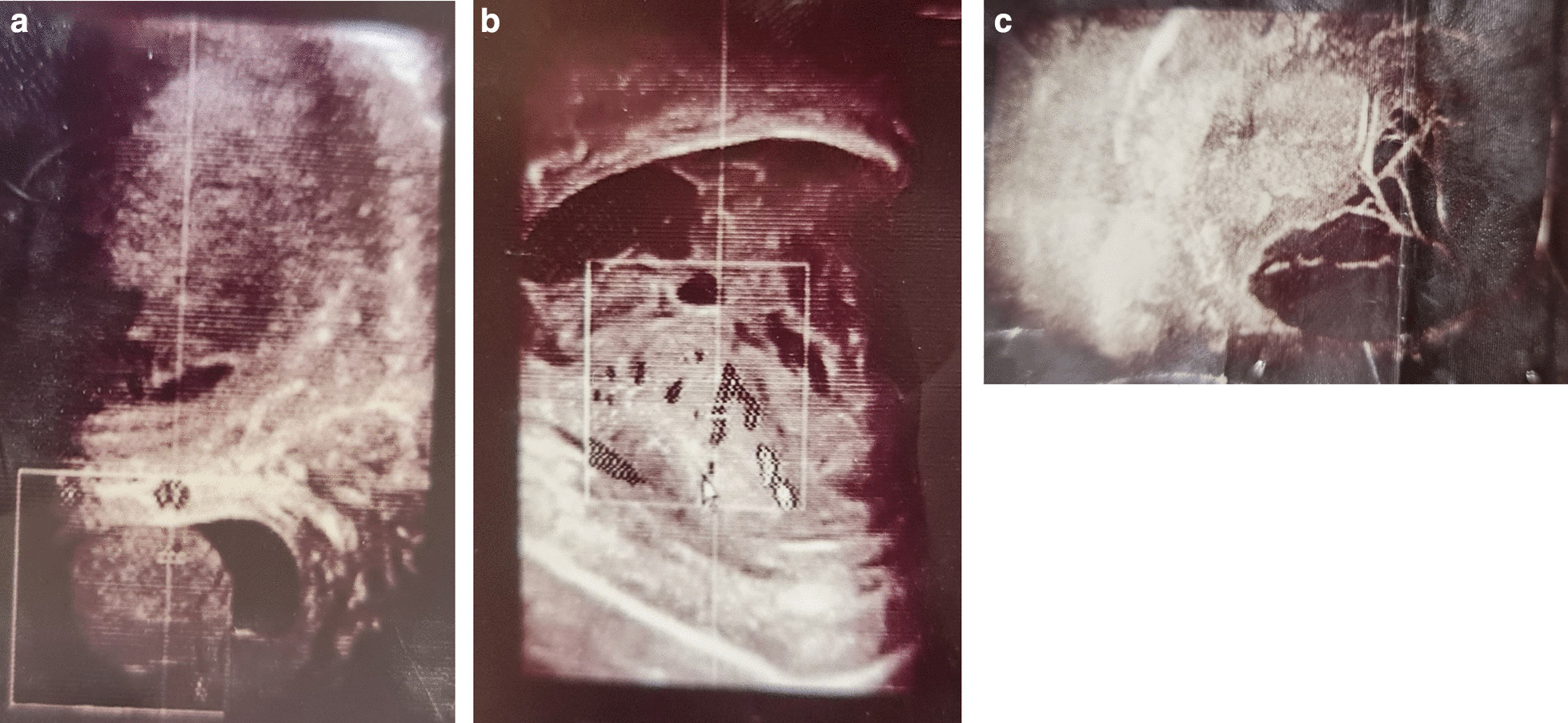
**A**–**C** Ultrasonographic examination of the testes (revealing a hyperemic and hypervascular right testicle and epididymis and a hydrocele with multiple septations)

Radiography was also performed using ultrasonography and chest X-ray (CXR). The obtained CXR revealed no abnormalities. Moreover, on abdominal ultrasonographic examinations, average dimensions and echogenicity of liver, gallbladder, pancreas, and spleen were noted with no abnormal masses. However, even though the parenchyma of the kidneys was found to be of normal echogenicity, mild left-sided hydronephrosis was reported. An inflamed mesenteric fat image in the right inguinal canal with air echoes in the distal canal was also observed, indicating an inguinal hernia. The ultrasonography of the scrotum revealed a hyperemic and hypervascular right testicle and epididymis (that is, unilateral epididymo-orchitis) and a hydrocele with multiple septations, suggesting the presence of either an abscess or hematoma (Fig. 2).

Moreover, the color Doppler ultrasound examination of the scrotum could not definitively rule out testicular torsion. Therefore, a surgery consult was requested, recommending surgical exploration to assess any ongoing scrotal and testicular pathology. In addition, scrotal hematoma or abscess and epididymo-orchitis were considered our differential diagnoses.

Upon surgical exploration, a normally sized right testicle was seen with no signs of torsion. However, a hydrocele sac was observed with reddish cloudy pus fluid, of which 5 mL was aspirated and sent for smear, component analysis, and culture. The smear results indicated a proteinous background, scattered neutrophils, lymphocyte infiltrations, and few mesothelial cells, while the culture indicated *E. coli* growth.

**Fig. 3:**
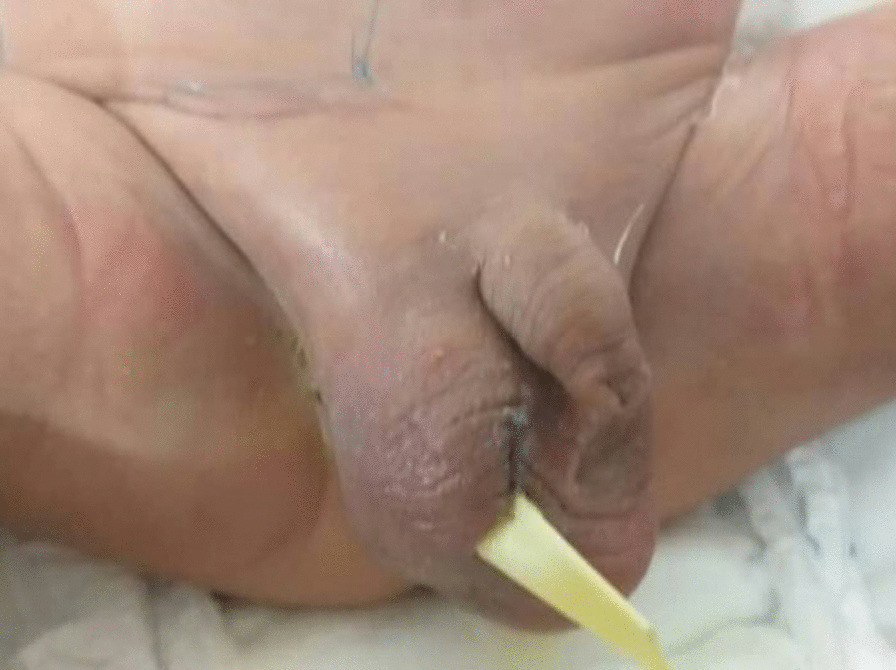
Scrotum appearance after surgical exploration

Subsequently, the antibiotic regimen initiated on admission was continued to treat the urinary tract infection until the culture returned without bacterial growth. Later, voiding cystourethrography (VCUG) was performed, and with its results indicating no anatomical and structural abnormalities, the patient was discharged on day 12 of admission completely recovered and in healthy condition (Fig. 3).

## Discussion

Considered an emergency, pyocele is a rare cause of acute scrotum in the neonatal and infantile period, having occurred in cases with varying ages, with testicular torsion ruled out as a differential diagnosis [2, 8]. The main suggestive features are clinical presentation indicating infection, including fever, scrotal and inguinal canal erythema, and tenderness [2]. Therefore, prompt treatment with broad-spectrum antibiotics is essential if detected early, with reports demonstrating favorable responses [2–4, 11].

Even though most cases of pyocele are idiopathic in etiology and the source of infection remains unrecognized, there have been descriptions of various possible mechanisms of pathogenesis in the literature [1, 6–8, 10]. These mechanisms include hematogenous seeding of the serosa lining, secondary infection of a hydrocele following orchitis or epididymitis, seeding from an intraperitoneal source through a patent tunica vaginalis, or reflux of urine into the vas deferens, which are the most common pathways at fault [3, 12, 13]. Moreover, the origins of some cases have been traced to maternal sepsis [14]. However, several cases of idiopathic pyocele have also been reported, with cultures from blood, urine, and other possible sources all returning negative for pathogenic growth [4, 14, 15].

In our case, the blood culture without any pathogen growth excluded the possibility of hematogenous seeding, while an abdominal ultrasonographic examination without abnormal findings excluded an intraperitoneal spread. Thus, an infection ascending from the urinary tract was the most probable route at fault due to urinary tract cultures indicating the growth of *E. coli*, with the culture of the drained fluid also returning with the same pathogen growth. Furthermore, surgical exploration, though unwarranted for the pyocele itself, was carried out to rule out the possibility of testicular torsion. However, previously published studies have demonstrated the effectiveness of both interventional and conservative approaches [2–4, 8–11, 14, 16–18]. For instance, one study reported the resolution of the infection with intravenous ampicillin and cefotaxime, with the former discontinued after obtaining the results from urine and blood cultures [3]. However, in one study on 56 newborns, 35 received only antibiotic therapy, while 21 underwent surgical exploration and drainage. Moreover, seven cases in the latter had an initial conservative management approach either due to unresponsiveness to the therapy or suffering from testicular necrosis [8]. These findings demonstrate that the physicians in charge of the patients’ treatment should thoroughly weigh the benefits against the risks of each of the mentioned approaches in a case-by-case manner and then decide on the most appropriate course of treatment.

## Conclusion

In cases presenting with fever and scrotal erythema and edema, testicular torsion and an infectious process should be considered, and amid ruling out the possibility of the former, cultures from all the susceptible bodily fluids, including blood and urine, as shown in this case, and the hydrocele itself, if warranted, and other evaluations are inconclusive, should be obtained to assess the latter. Moreover, while awaiting the results, empirical antibiotics should be prescribed to treat possible underlying sepsis.

## Data Availability

The dataset supporting the conclusions of this article is available upon request to the corresponding author.

## Abbreviations

*E. coli*: *Escherichia coli*
CXR: Chest X-ray
mg/kg: Milligrams per kilograms
VCUG: Voiding cystourethrography
bpm: Beats per minute
Neut: Neutrophils
Lymph: Lymphocytes

## References

[1] Basta AM, Courtier J, Phelps A, Copp HL, MacKenzie JD (2015). Scrotal swelling in the neonate. J Ultrasound Med.

[2] Oberlin DT, Cheng EY (2015). Management of pediatric pyocele using percutaneous imaging-guided aspiration. Int J Surg Case Rep.

[3] Aguilera-Alonso D, Del Rosal T, Pérez Muñoz S, Baquero-Artigao F (2018). Neonatal epididymo-orchitis with pyocele caused by *Escherichia coli*: successful treatment with antimicrobial therapy alone. Enferm Infecc Microbiol Clin (Engl Ed).

[4] Kraft KH, Lambert SM, Snyder HM, Canning DA (2012). Pyocele of the scrotum in the pediatric patient. J Pediatr Urol.

[5] Kim SH, Cho YH, Kim HY, Lee N, Han YM, Byun SY (2021). Scrotal pyocele secondary to gastrointestinal perforation in infants: three case reports. Yeungnam Univ J Med..

[6] Santucci RA, Krieger JN (1995). Pyocele of the scrotum: a consequence of spontaneous bacterial peritonitis. J Urol.

[7] Omran A, Gawrieh BS, Abdo A, Ali Deeb M, Khalil MA, Shater W (2019). Amyand hernia: scrotal pyocele, associated with perforated vermiform appendix and complicated by testicular ischemia in neonate. J Surg Case Rep.

[8] He TQ, Zhu LH, Li CY, Peng QL, Zu JC, Liu Y, Zhao YW (2022). Clinical analysis of pyocele of tunica vaginalis in 56 newborns. Urol Int.

[9] Asif AA, Shah N, McBeeOrzulak FJ (2021). Staphylococcus lugdunensis causing epididymo-orchitis with scrotal pyocele. IDCases.

[10] Ramjit A, Shin C, Hayim M (2020). Complete testicular infarction secondary to epididymoorchitis and pyocele. Radiol Case Rep.

[11] Bruner DI, Ventura EL, Devlin JJ (2012). Scrotal pyocele: uncommon urologic emergency. J Emerg Trauma Shock.

[12] Chiang M-C, Chen H-W, Fu R-H, Lien R, Wang T-M, Hsu J-F (2007). Clinical features of testicular torsion and epididymo-orchitis in infants younger than 3 months. J Pediatr Surg.

[13] Halachmi S, Katz N. Epididymo-orchitis in pre-pubertal children. Epidemiology, etiology, management and follow-up recommendations. Open J Urol. 2013;3(2):96–101.

[14] Terentiev V, Dickman E, Zerzan J, Arroyo A (2015). Idiopathic infant pyocele: a case report and review of the literature. J Emerg Med.

[15] Hsieh DS, Jeng SY, Liu YS (1998). Bilateral idiopathic infantile pyoceles: a case report. Zhonghua Yi Xue Za Zhi (Taipei).

[16] Chiang MC, Wang TM, Fu RH, Chu SM, Chou YH (2005). Early-onset *Escherichia coli* sepsis presenting as acute scrotum in preterm infant. Urology.

[17] Kim SH, Cho YH, Kim HY, Lee N, Han YM, Byun SY (2023). Scrotal pyocele secondary to gastrointestinal perforation in infants: a case series. J Yeungnam Med Sci.

[18] Mondal N, Sharma S, Balachander B, Vaishnav D, Plakkal N, Vishnu BB (2016). Neonatal sepsis presenting as pyocele. Indian J Pediatr.

